# Supplementary material for: A modified fluctuation-test framework characterizes the population dynamics and mutation rate of colorectal cancer persister cells
Source: Nat Genet. 2022 Jul 11;54(7):976–84. doi: 10.1038/s41588-022-01105-z (PMC9279152; doi:10.1038/s41588-022-01105-z)
Supplement: Supplementary file 1 — Supplementary Note 1. Supplementary results. Supplementary Note 2. Supplementary methods. Supplementary Note 3. Supplementary tables. Supplementary Note 4. Supplementary figures and legends. Supplementary Note 5. Supplementary references. [file 41588_2022_1105_MOESM1_ESM.pdf]

nature portfolio

https://doi.org/10.1038/s41588-022-01105-z

Supplementary information

A modified fluctuation-test framework characterizes the population dynamics and mutation rate of colorectal cancer persister cells

In the format provided by the authors and unedited

Supplementary Information

A modified fluctuation-test framework characterizes the population dynamics and mutation rate of colorectal cancer persister cells

Mariangela Russo[1,2,*], Simone Pompei[3,*], Alberto Sogari[1,2,*], Mattia Corigliano[3,4,*], Giovanni Crisafulli[1,2], Alberto Puliafito[1,2], Simona Lamba[2], Jessica Erriquez[2], Andrea Bertotti[1,2], Marco Gherardi[3,4], Federica Di Nicolantonio[1,2], Alberto Bardelli[1,2, †], and Marco Cosentino Lagomarsino[3,4,†]

[1]Department of Oncology, University of Torino, Candiolo (TO), Italy.

[2]Candiolo Cancer Institute, FPO–IRCCS, Candiolo (TO), Italy.

[3]IFOM Foundation, FIRC Institute of Molecular Oncology, Milan, Italy.

[4]Dipartimento di Fisica, Università degli Studi di Milano, and I.N.F.N, via Celoria 16 Milano, Italy

[*] These authors contributed equally.

† These authors jointly supervised this work.

Correspondence:

Alberto Bardelli (alberto.bardelli@unito.it)

Marco Cosentino Lagomarsino (marco.cosentino-lagomarsino@ifom.eu)

Supplementary Note 1: Supplementary Results

Supplementary Note 2: Supplementary Methods

Supplementary Note 3: Supplementary Tables

Supplementary Note 4: Supplementary Figures and Legends

Supplementary Note 5: Supplementary References

Supplementary Results

Growth dynamics of CRC cells in the absence of drug-treatment.

With the aim of calculating whether drug treatment affects the mutation rate of tumor cells, we first designed a set of biological experiments to infer growth dynamics parameters of CRC cell populations. These included: (i) growth rates in standard cell culture conditions; (ii) population dynamics under treatment with targeted therapies; (iii) population dynamics of residual persister cells. To characterize the population of cancer persister cells that emerges in response to targeted therapies we studied two microsatellite-stable (MSS) CRC cell lines, for which we previously found evidence for the emergence of persisters[1]. DiFi cells carry amplification of the *EGFR* gene and are *RAS/RAF*-wildtype, which renders them highly sensitive to the anti-EGFR antibody cetuximab, paralleling the subset of CRC patients responsive to EGFR blockade [1,2]. As a second model we used *BRAF* V600E-mutated WiDr cells, which are sensitive to BRAF inhibition in combination with cetuximab [1], a therapeutic regimen recently introduced for the treatment of metastatic CRC patients with *BRAF* mutations [3,4]. These cell models were established before the clinical development of anti-EGFR and BRAF targeted therapies in CRC, ruling out the possibility that drug exposure could have occurred in the patients from whom the cells were derived [5].

To reduce the possibility that pre-existing resistant cells were present in the populations at the beginning of the assays, we first isolated individual clones for each cell model, with growth and drug sensitivity profiles comparable to those of the parental population from which they originated (Supplementary figure 1a and b, respectively).

We first measured birth and death rates of clones in standard cell culture conditions. We defined WiDr and DiFi population dynamics by a standard birth-death process with two parameters: the birth rate b, i.e., the rate at which new cells are generated through cell division, and the death rate d. For each cell model, we measured daily the total number of viable and dead cells (Supplementary figure 2a). The effective growth rate ($b - d$) was estimated with an exponential fit of the growth curve of each clone (Supplementary figure 2b and Supplementary table 1); while the value d/b was estimated from the fraction of dead cells detected by flow cytometry (Supplementary figure 2c and Supplementary table 1; see Supplementary Methods below for mathematical details). Finally, the values of the birth and death rate in the absence of treatment were obtained by combining the two estimates (values of b-d and d/b) (Supplementary table 1).

Growth dynamics of CRC cells under drug-treatment.

The population dynamics of cancer cells during drug treatment involve both growth in presence of the drug and the transition of sensitive cells to a persister phenotype. The transition to persisters can be described as follows: each cell has the possibility to reversibly turn into persister at a certain rate, which could depend on external conditions (e.g., drug concentration). Growth/division parameters of persister cells, as well as their death rate under drug treatment, differ from those of the original sensitive population (see below for a mathematical formulation of these rules). To characterize the emergence of the persister subpopulation, and to quantify the intertwined processes of growth and transition to persister state upon drug treatment, we collected data from two sets of drug-response growth assays (Fig. 1a). In the first one (referred to as '*doses-response assay*'), we evaluated response of the two cell models to multiple doses of targeted therapies (Fig. 1a). Both DiFi and WiDr clones were exposed to increasing concentrations of the EGFR inhibitor cetuximab alone or in combination with the BRAF inhibitor dabrafenib, respectively. Cell viability of

WiDr cells was assessed daily over a 5-day treatment period. Since we observed a slower decay in the DiFi cell number during cetuximab treatment, we extended measurements to 19 days in this model (Fig. 1a, 1b, Extended data fig.1a). Data from *doses-response assay* were used to analyze growth curves defined as number of live cells vs time and concentration of drug treatment (Fig. 1c, Extended data fig.1b). The normalization process of the *doses-response assay* data used to obtain growth curves is illustrated in Supplementary figure 4 (see Supplementary Methods).

We then assessed response to a constant drug concentration by analyzing the fraction of surviving cells over 3 weeks of treatment (referred to as '*single-dose assay*') (Fig. 1a and d). These results highlight a biphasic two time-scale exponential decay (Fig. 1d, Extended data fig. 1c): when observed in bacteria, this pattern is considered a characteristic of the emergence of persister sub-populations [6]. We took this as a proof of the emergence of persisters, and defined persisters as the residual sub-populations showing a very-slow death rate upon continuous treatment. Both DiFi clones and WiDr cl. B7 displayed a slow but measurable decay in cell number, compatible with an exponential decay, suggesting a tendency of persisters to slowly die over time, while a stable plateau was observed for WiDr cl. B5 (Fig. 1d, Extended data fig. 1c and Extended data fig. 2).

CRC persister cells slowly replicate during drug treatment

Persisters are capable of surviving the lethal effect of the drug by entering a non-replicating or slowly replicating state [7]. We and others have recently reported that drug-treated cancer cells, akin to bacteria exposed to antibiotics, initiate adaptive mutability stress response [1,8], a process likely involving cell division and DNA replication. We therefore set out to establish whether persister cells divide under drug pressure. We treated CRC cell models with the corresponding targeted therapies for two weeks, until cells reached the persister state, and then stained the residual persister cells

with Carboxy fluorescein succinimidyl ester (CFSE), a cell-permeable fluorescent dye allowing flowcytometric monitoring of cell divisions. Upon passive diffusion through the cell membrane of viable cells, CFSE is cleaved by intracellular esterases and retained within their cytoplasm. When a cell divides, CFSE is uniformly segregated between daughter cells; therefore, the reduction of CFSE intensity is proportional to the number of cell divisions [9].

CFSE-labelled DiFi and WiDr cells were analyzed by flow cytometry at different time points. To discriminate between persisters and pre-existing resistant cells, we seeded and treated each cell model in multiple 24-multiwell plates and excluded from the analysis wells containing clones that grew in two weeks in the presence of the drug. The analysis unveiled that persister cells slowly replicate during treatment (Extended data fig. 3a).

We next quantified the fraction of proliferating persisters by fluorescently labeling cell surviving to 2 weeks of constant drug treatment with EdU (5-ethynyl-2'-deoxyuridine), a modified thymidine analogue that is efficiently incorporated into DNA during active DNA replication. Quantification of EdU positive cancer persister cells after 14, 17 and 20 days of drug exposure unveiled a fraction between 0.2 and 2.5 % of replicating persisters depending on the cell model (Extended data fig. 3b, c). These findings are in line with recent data showing a fraction of cycling persister cells emerging during treatment of lung cancer cells with an EGFR inhibitor[10].

To better elucidate dynamics of persister cell proliferation and death mechanisms under drug treatment, we set up a live cell microscopy imaging assay. Both DiFi and WiDr clones were treated for 2 weeks with the anti-EGFR antibody cetuximab alone or in combination with BRAF inhibitor dabrafenib, respectively, until the emergence of persister cells. We then labeled surviving persister cells with live fluorescent dyes allowing accurate and sensitive detection of cell divisions (through

nuclear fluorescent labeling with Nucblue®) and cell death (through CellEvent®, a live fluorescent dye to detect Caspase3/7 activation) within persisters subpopulation across several days of drug treatment.

While the majority of CRC persisters were non-replicating cells, we unveiled cell division events in all the CRC clones analyzed (Extended data fig. 3d and Supplementary Movies 1-4). In some instances, the cell division was successful and vital (Extended data fig. 3d), while in others the cells died concomitantly (Extended data fig. 3e). In parallel, we detected cell death, as highlighted by cells showing positive stain for green fluorescent light either after a cell division event, either in non-dividing cells (Extended data fig. 3e and f, respectively). Data from the imaging assay was also used to estimate (approximately, due to the small statistics) the effective growth rate of persister cells, which is compatible with the values obtained by the TP model inference (Extended data fig. 3g). Quantification of net growth rate of persister cells unveiled a slow decay in cell number, supporting a balance between proliferation and cell death slightly in favor of the latter.

Supplementary Methods

Theoretical Modeling

Deterministic and stochastic model. In this study we have developed and used two distinct mathematical models to investigate the dynamics of cell populations. The first model describes the transition to persister state ("TP model"), and is a birth-death model with phenotypic switching, which we explored in the deterministic limit, i.e., neglecting statistical fluctuations and only considering expected values of the model output. We made use of this model for two purposes: (i) to infer the parameters of persisters dynamics, and (ii) for the Bayesian model-selection inference procedure that was used to infer whether the transition to persisters is drug-induced or not. This deterministic model is described in the sections *Dynamics of sensitive (untreated) cells* and *Dynamics of cell population under drug treatment* below.

The second model, which we named *Mammalian Cells-Luria Delbrück* or "MC-LD" model, is a fully stochastic birth-death branching process that includes the mutational processes of sensitive (untreated) and persister cells (under treatment). In order to measure the mutation rate, stochastic fluctuations cannot be neglected. Therefore, we considered this extension of the TP model including (i) stochastic fluctuations around the expected values of the deterministic limit and (ii) the mutational processes of both sensitive and persister cells. Relatedly, we did not include the latter process in the TP model, since during first days of drug treatment death of sensitive cells or transition to persisters are predominant, while acquisition of mutations driving resistance becomes significant only after weeks of treatment.

We have derived an approximate solution for the fraction of resistant wells in the MC-LD model, to derive estimators of mutation rate, as described in the Supplementary methods section *Inference of the mutation rate from a two step fluctuation assay* below, and we have run the model by direct

simulation in order to design the biological experiments and validate the estimators that we obtained (Fig. 4a, c). Simulations of this model were also used to investigate the distribution of the number of persister cells after 3 weeks of treatment (Extended data fig. 6), by setting to zero the value of the mutation rates.

Dynamics of sensitive (untreated) cells. The drug sensitive clonal cell populations were modelled with a standard birth-death process. Their dynamics is therefore described by two parameters: (i) the birth rate (*b*) (i.e., the rate at which new cells are generated by replication) and (ii) the death rate (*d*). This model describes an exponential growth for the number $N_s(t)$ of viable cells at time *t*:

$$N_s\,(t) = N_s(0)\; e^{(b-d)t} \qquad (1)$$

where $N_s(0)$ is the number of viable cells present at *t* = 0. Another dynamical variable which can be measured from the flow cytometry analysis (Supplementary figure 2c) is the fraction of dead cells δ which, at equilibrium, takes the value

$$\delta = \frac{d}{b} \qquad (2)$$

Hence, an indirect estimate of *b* and *d* can be obtained in two steps: (i) an exponential fit of the observed growth curves (Eq. 1) gives the value *b* − *d*; (ii) an estimate of the asymptotic fraction of dead cells (Eq. 2) gives the value *d/b*. We do not model explicitly the possibility of reversible switching to persister state in absence of treatment, as this process is not observable in our experimental data. However, the model accounts for this process via the parameter f_0, the fraction of persister cells at the time of treatment administration (see below).

Dynamics of cell population under drug treatment. We focus on the dynamics of a population of $N(t) = X(t)+Z(t)$ total viable cells, consisting of a combination of $X(t)$ sensitive cells and $Z(t)$ persister cells. The population is assumed to grow in presence of a drug with constant drug concentration

[M]. Treated sensitive cells can (i) reproduce with a birth rate B, (ii) die with a drug-dependent death rate $D([M])$, (iii) switch to the persister state with a drug-dependent transition rate $\lambda([M])$.

Persister cells display a moderate division rate under drug treatment (Extended data fig. 3), and their observed dynamics under long-term treatment indicates a slow decline in cells number (Fig. 1d) compatible with a negative, but small, effective growth rate. These two observations can be described by a scenario where persister cells that attempt to divide before developing drug-resistance mutations die out (i.e., for which the net growth rate is $\simeq 0$), while non-dividing persister cells slowly die during the treatment. In this scanario back-switching from persister to sensitive in presence of the drug effectively enters the model as a contribution to death rate, and this combined dynamics are described by the effective death rate $D_p > 0$.

Drug effect is assumed to be delayed by a time t_0 after the administration of the drug to the cancer cell population. During this time interval, the model assumes that the cell popoulation grows with a net growth rate S_0:

$$N(t) = N_0\, e^{S_0 t} \qquad t \leq t_0 \tag{3}$$

Where N_0 is the initial number of cells. The quantity $f_0 = \frac{Z(t_0)}{N(t_0)}$ is the fraction of persister cells present at the time t_0, i.e., at the time of the effective initiation of the drug effect. This parameter effectively incorporates the possibility of pre-existence of persisters due to reversible switching in absence of the drug. Under these assumptions, the dynamics of the fraction of sensitive cells during treatment is described by

$$x(t) = \frac{X(t)}{N_0} = (1 - f_0)\, e^{S_0 t_0 + S([M])(t - t_0)} \tag{4}$$

and we have defined $S([M]) = B - D([M]) - \lambda([M])$ as the net growth rate of sensitive cells under drug treatment. Similarly, the fraction of persister cells during the treatment is described by

$$z(t) = \frac{Z(t)}{N_0} = f_0\, e^{S_0 t_0 - D_p(t-t_0)} + (1 - f_0)\, e^{S_0 t_0} \frac{\lambda([M])}{S([D]) + Dp} \left(e^{S([M])(t-t_0)} - e^{-D_p(t-t_0)}\right). \qquad (5)$$

The quantity

$$n(t) = \frac{N(t)}{N_0} = \begin{cases} e^{S_0 t} & t \leq t_0 \\ x(t) + z(t) & t > t_0 \end{cases} \qquad (6)$$

was used to “learn” the model and its parameters from the experimental data (see below).

Death rate of treated cells. The death rate of sensitive cells during drug treatment is assumed to increase from its basal level ($D[0] \equiv D_0$) due to the effect of the drugs. In particular, based on previous evidence [15] , we model this dependence as

$$D([M]) = D_0 + k_0\left(1 - e^{-a\,[M]}\right) \qquad (7)$$

where k_0 is the maximum increase of death rate due to the drug effect. The parameter a^{-1} sets a characteristic drug concentration: for $[M] \ll a^{-1}$ cells die with their unperturbed (basal) death rate D_0, while for concentrations $[M] \gg a^{-1}$ the death rate reaches the maximal value $D_0 + k_0$. The empirical form Eq. (7) describes an exponential interpolation between these two extreme cases.

Transition rate to persistence. We considered four possible model variants for $\lambda([M])$: (i) a null model with no persistence state, corresponding to the case $\lambda = 0$ for any value of the drug concentration; (ii) a model with a drug-independent transition rate $\lambda = \lambda_0$; two models where the transition rate is linearly dependent on drug concentration, (iii) $\lambda([M]) = k[M]$ and (iv) $\lambda([M]) = k[M] + \lambda_0$. This latter functional dependence should be considered as the first term expansion of a non-

linear model with a rate saturating at high [M] values. The four model variants are associated to clearly distinguishable patterns as summarised in Extended data fig. 4.

Drug-dependent transition rate and minimization of number persister cells. The best TP model variant that describes the observed population dynamics for WiDr cells is the one with a transition rate to persistence that is linearly dependent on drug concentration $\lambda([M]) = k\,[M]$ (see below). This particular functional dependence corresponds to a scenario where higher drug concentrations induce both an increased death rate of sensitive cells, which is desirable, but, at the same time, an increased transition to the persister state, which will act as a reservioir for the emergence of resistance. Here we show that, by modulating the drug concentration over time, this trade-off can potentially be exploited to reduce the number of persister cells emerging in response to the therapeutic drug.

To illustrate this point we considered two alternative drug delivery strategies: (i) the concentration of the drug is kept constant over time (Extended data fig. 5a) vs (ii) the concentration of the drug is linearly increased over time (Extended data fig. 5b). In order to keep the two strategies comparable, this second strategy is constrained to have the same average drug concentration as the first, meaning that the total amount of drug delivered is the same for the two cases. The choice of the two strategies is shown in the Extended data fig. 5a,b.

We solved the TP model (eq.1) numerically, using these two alternative choices for the dependence of the drug concentration over time as model inputs, and compared the two outcomes (Extended data fig. 5c). The other model parameters were set to the values inferred for the case of WiDr cl. B7 (see Supplementary table 4). In order to investigate the worst case scenario, i.e. the one with the maximal number of emergent persister cells, we neglected their death (D_p=0). We found that a drug

delivery strategy with a linearly increasing drug concentration can actually reduce the total number of emergent persister cells (Extended data fig. 5d).

Computer Simulations for the stochastic modeling. We simulated individual trajectories of the Markov process underlying the evolution of the MC-LD model. A well-known exact algorithm to simulate individual trajectories of a Markov process was provided by Gillespie [16]. However, this algorithm was too slow for the models and population sizes considered in this study. Therefore, we used a coarse-grained version of the algorithm, which groups together all stochastic events happening in discrete time intervals of fixed duration Δt. This approximation is equivalent to assuming that events are independent within the time scale of Δt. We adjusted the parameter Δt so as to minimize the errors, while still keeping the simulations numerically feasible. Sensitive cells were assigned values of the effective proliferation parameters estimated from data under treatment $B - D[M]$ (values reported in Supplementary table 2), and values of the division rate $B \simeq b/2$, where b is the division rate of untreated sensitive cells (see Supplementary table 1). Persister cells were given effective proliferation parameters $b_p = 0$ (birth rate) and $d_p = D_p$ (death rate). It should be noted that the form of the estimator used to infer the mutation rate of persister cells (Eq 15) only depends on the difference $b_p - d_p = -D_p$ and $B - D[M]$. Hence, to test the validity of our methods, the actual numerical value of the division rate did not matter. Persister cells turn into a resistant cell with a chronological rate μ_p (per cell, per unit of time). Once a persister cell has turned into a resistant one, it immediately transitions to a cell whose proliferation rates correspond to those of untreated sensitive cells (Supplementary table 1).

Inference of parameters describing the cell population dynamics

Birth and death rates of sensitive cells. For the inference of the birth-death rates *b* and *d*, we used the data set described in the Methods section *Growth rates of CRC clones before drug treatment*, together with the model described in the Supplementary methods section *Dynamics of sensitive (untreated) cells* (above). Our inference scheme is summarized in Supplementary figure 2a. The net growth rate *b−d* was estimated with an exponential fit against the observed growth curve of the two clones (WiDr and DiFi) in a time span of 4 days starting from 40x10^4 cells/well in a 6-multiwell plate (Supplementary figure 2b). The value *d/b* was estimated from the observed asymptotic fraction of dead cells detected by flow cytometry analysis (Supplementary figure 2c). Finally, the values of the birth and death rate of sensitive cells were obtained by combining the two estimates (value of *b − d* and *d/b*). Estimated values are reported in Supplementary table 1.

Calculation of growth curves from drug response assays. Growth curves of CRC clones under treatment, reported as fold-change of viable cells *vs* time of drug exposure, were calculated from the *doses-response assay* (Figs 1c and 2b; Extended data fig. 1b, 8a and 9a) and from the *single-dose assay* (Figs 1d and 2c; Extended data fig. 1c and 8b). We used the following strategy. Both data-sets consisted in measurements of cell viability (luminescence signal) with constant drug concentraction ($[M]$), evaluated at several time points ($t = 1,2,..,t_{max}$ days) and for a set of biological replicates ($i = 0,1,..,n$). We denote with $Y_t^i([M])$ the value of cell viability of the ith biological replicate, of a drug-response growth assay performed with drug concentration $[M]$, and evaluated at time t.

These ATP measurements present a day-to-day variability, which is reflected in a variability across replicates, but also present regularity in their behavior, which we aimed to extract. Specifically, the curves of different replicates look similar, but are affected by offsets that vary from day to day.

Hence, we first normalized data corresponding to different biological replicates of the same clone in the following way. The values $Y_t^i([M])$ were multiplied by a normalization factor c^i, the same for all the data collected in the same biological replicate, but different across replicates. These normalization factors were obtained by minimizing the total relative error across replicates $ER = \sum_{[M]} \sum_t \frac{\sigma^2([M],t)}{\mu^2([M],t)}$, where $\mu([M],t) = \frac{1}{n}\sum_{i=1}^{n} c^i Y_t^i([M])$ and $\sigma^2([M],t) = \frac{1}{n}\sum_{i=1}^{n}\left(c^i Y_t^i([M]) - \mu([M],t)\ \right)^2$ are the average and standard deviation of the viability normalized with weights c^n across replicates, evaluated at time t and for drug concentration $[M]$. The effect of this normalization process is illustrated in the Supplementary figure 3.

Average values and standard deviation computed with the optimal values of the normalization factors obtained by minimizing ER were then divided by the average viability measured at day 0 ($\mu([M], t = 0)$) to obtain growth curves: y([M],t) $= \frac{\mu([M],t)}{\mu([M],t=0)}$ with associated standard deviation $\sigma_y(\mathrm{[M]}, \mathrm{t}) = \frac{\sigma([M],t)}{\mu([M],t=0)}$.

TP model variants. Growth curves of WiDr and DiFi clones were used to infer model parameters of 8 (4 X 2) distinct model variants, i.e., configurations of the TP model with different assumptions of the two key model parameters: (i) the rate at which sensitive cells switch to persisters during the treament (λ) and (ii) the initial fraction of persister cells (f_0). More in detail, we considered 4 model variants associated to the choice of the transition rate $\lambda([M])$ = $\{0, \lambda_0, k[M], k[M] + \lambda_0\}$ which we combined with 2 variants using different ranges of the numerical values of the intial fraction of persisters ($f_0 = 0$ and $0 < f_0 < 1$).

Inference of the TP model parameters. The parameters of the TP model (Eq. 6), with all the 4 model variants for the transition rate $\lambda([M])$ = $\{0, \lambda_0, k[M], k[M] + \lambda_0\}$ and the two variants for the initial

fraction of persisters ($f_0 = 0$ and $0 < f_0 < 1$), have been inferred using a Bayesian framework. For the inference of TP model parameters in WiDr we used growth curves assessed from both the *doses-response assay* and the *single-dose assay*, while for DiFi we used only growth curves assessed from the *doses-response assay*. Posterior distributions of the model parameters were sampled using a Hamiltonian Monte Carlo (HMC) algorithm (*Python 3*, package *pymc3*, NUTS sampler) [17]. The likelihood function was set to the product of standard Gaussian likelihood functions over the observed data points, with parameters equal to the mean value and standard deviation of the data points. We assumed flat prior distributions of the model parameters; the corresponding supports, i.e. maximum and minimum values allowed, are reported in the Supplementary table 2. For WiDr, model fit was performed including all the growth curves evaluated with dabrafenib concenteration $[M] \geq 0.041\ \mu M$, while for DiFi we included all growth curves evalutaed with cetuximab drug concenteration $[M] \geq 2.1\ nM$. Value of the model parameters describing the drug delay in the *single-dose assay* for DiFi ($t_0(single), S_0(single)$) were inferred with an independent model fit, while keeping all the other parameters fixed (the remaining parameters were inferred from the growth curves derived from the *doses-response assay*). Values for these two parameters are reported in the Supplementary table 2. For DiFi cl. B3, the doses-response dataset already displayed the bi-phasic behavior (see Extended data fig. 1), and this data-set alone was sufficient to extract all the TP model parameters. For the inference of the model parameters of this clone, we set the values (t_0, S_0) to the maximum value observed.

Comparison between TP model variants. The logic flow of the comparison between model variants within our inference scheme is summarized in Extended data fig. 4. The eight TP model variants (2 choices of f_0 for each of the 4 λ models) were compared by means of the standard Bayesian Information Criterion (BIC) and the Akaike Information Criterion (AIC) (Supplementary table 3).

These quantities measure model performance keeping into account the number of parameters used (penalizing model variants with more parameters). We found that both the AIC and BIC indicate that the drug-induced scenario ($f_0 = 0$) is the preferred variant for all the cell lines (Supplementary table 3 and Extended data fig.4).

In addition, we found that the best λ model for WiDr is the one where the transition rate to persistence is linearly proportional to the drug concentration $\lambda([M]) = k[M]$, while for DiFi the data is best described by a TP model variant with a constant transition rate $\lambda([M]) = \lambda_0$ (Supplementary table 3 and Extended data fig.4). Of note, the two functional dependences observed for WiDr and DiFi clones can be recapitulated by the following function

$$\lambda([M]) = \lambda_0\left(1 - e^{-\beta\,[M]}\right)$$

This particular form is indeed compatible with both a constant value, which is reached for high values of the concentrations:

$$[M] \gg \beta^{-1} \rightarrow \lambda([M]) = \lambda_0$$

and with a linear increase, attained for values

$$\beta * [M] \ll 1 \rightarrow \lambda([M]) \simeq \lambda_0 * \beta * [M] \equiv k * [M]$$

Single dose data of WiDr cl. B7 are therefore compatible with this functional form for values of $\beta^{-1} > 10^{-4}$ Mol, although it is not possible to identify the exact value of this model parameter because it is probably bigger than the highest concentration we consider (we do not see any signal for the saturation of the transition rate). Conversely, the single dose data of DiFi cl. B6 is compatible with this functional dependence for values of $\beta^{-1} \geq 10^{-8}\ [Mol]$ and of $\lambda_0 = 0.234\ days^{-1}$ (see Supplementary figure 6).

Hence, the two clones have a dependence on the drug concentration that can be explained by the same functional dependence, but with different values of the typical inverse concentration β. We

speculate that this difference is related to the fact that the two clones are sensitive to a different drug combination (cetuximab + dabrafenib for WiDr, cetuximab alone for DiFi).

Choice of the TP model variant for the inference of the mutation rate. The parameters used to calculate the mutation rates were inferred using the following TP model variants: (i) transition rate to persistence linearly proportional to the drug concentration $\lambda([M]) = k[M]$ and $0 < f_0 < 1$ for WiDr cells and (ii) constant transition rate $\lambda([M]) = \lambda_0$ and $0 < f_0 < 1$ for DiFi cells. For both clones, the choice of the best λ model was informed by the BIC and AIC indices (Supplementary table 3), while in both cases we made use of the variant with $0 < f_0 < 1$ even though $f_0 = 0$ was preferred by the BIC and AIC indices (Supplementary table 3). This was done in order to have a realistic estimate of the maximum value of f_0 which is still compatible with the experimental growth curves data. In this way we were able to infer the value of the mutation rate taking into account the effect of a fraction of persisters that could have been present in the population before drug administration. The fit of these models to the experimental data is shown in Fig. 2 and Extended data fig. 2 while the statistics of the corresponding model parameters are reported in Supplementary table 4. Corresponding posterior distributions are shown in Supplementary figure 5. Plots of the posterior distributions were obtained with the software GetDist [18]. Note that the approach used in Figs 2d and 4d and discussed in the main text does not focus only on the optimal model variant (i.e., $f_0 = 0$), but explores all the possible values of f_0.

Distribution of persister cell abundance. The TP model presented in the previous section was extended to include stochastic effects to investigate the distribution across wells of the number of persister cells at a given time point (well-to-well number variability). To this aim, we made use of computer simulations (Supplementary methods section *Computer Simulations for the stochastic*

modeling). We found that in the drug-induced scenario, i.e., in the absence of persister cells before drug treatment ($f_0 = 0$), and for time of drug treatment $\gg 1\ day$, the number of cells is distributed according to a Poisson distribution (see Extended data fig. 6). Extended data fig. 6b exploits this relation to verify that the drug-induced scenario is compatible with the observed dynamics of both CRC cell clones, using the experimentally measured well-to-well distribution of cell numbers measured after 3 weeks of treatment. The cumulative distribution of the cell viability measurements across well of drug-tolerant persister cells is indeed compatible with a Poisson cumulative distribution (Extended data fig. 6b). Since the ATP assay measures the number of cells through an unknown constant of proportionality between the ATP content ad the actual number of viable cells, in order to fit the observed distribution of ATP assay readouts to a Poisson distribution we proceeded as follows. We computed the value $\alpha = \sigma_{ATP}^2/\mu_{ATP}$, i.e., the ratio of the variance (σ_{ATP}^2) over the mean (μ_{ATP}) of the ATP measurements across the wells. The best fit to the distribution of the ATP measurements was the function $C\left(\frac{\mu_{ATP}}{\alpha}, ax\right)$, where $C(\mu, x)$ is the cumulative distribution function of a variable (x) distributed according to a Poisson distribution with mean μ.

Inference of the mutation rate from a two step fluctuation assay

Probability for the emergence of at least one mutant. This section derives from the MC-LD model an approximate expression for the probability of the emergence of one mutant in an expanding population of cells in a given time interval [0,*T*]. In the MC-LD model, we denote with N(*t*) the number of viable cells present in the population at time *t*, and with μ the effective rate at which one individual becomes a mutant. We assumed that mutant individuals in the population have the same dynamical rates as untreated cells, i.e., they divide with rate *b* and die with rate *d*. Because of reproductive fluctuations (genetic drift), cells carrying drug-resistance mutations can still go extinct, and only a fraction of the mutants will “establish” in the population, and survive. We refer to these

cells in the following as "established mutants", using the standard terminology of population genetics.

The probability of surviving stochastic drift in a time interval Δt is a well-known result of the birth-death process [19,20], which reads

$$\psi(\Delta t) = \frac{(b-d)e^{(b-d)\Delta t}}{b\, e^{(b-d)\Delta t} - d} \tag{8}$$

With these assumptions and up to the first order in μ (i.e., assuming a sufficiently small mutation rate) the expected number of emerging mutants establishing in $[0,T]$ is given by

$$\mathcal{M}(T) = \mu \int_0^T N(t)|_{\mu=0}\, \psi(T-t)dt + \mathcal{O}(\mu^2) \equiv \mu\mathcal{N}(T), \tag{9}$$

where we have defined $\mathcal{N}(T) = \int_0^T N(t)|_{\mu=0}\, \psi(T-t)dt$. The number of established mutant cells is Poisson distributed with expected value $\mathcal{M}(T)$ and consequently the probability of having at least one mutant is given by

$$P(T) = 1 - e^{-\mathcal{M}(T)}\,. \tag{10}$$

In the context of the fluctuation test, this probability is estimated by $\hat{P}$, i.e., the fraction of wells that have developed resistant mutants by the time T after treatment. The generalized estimator of the mutation rate then takes the form

$$\hat{\mu} = -\frac{\log\left(1-\hat{\mathrm{P}}\right)}{\mathcal{N}(\mathrm{T})}\,. \tag{11}$$

This general form of the estimator is valid for both pre-existing and persisters-derived resistant cells, and has been tested with synthetic data (see Fig. 4c). More specific expressions that can be used with data are found below.

Estimator of mutation rate for sensitive cells. As in a standard fluctuation test, to estimate the spontaneous mutation rate of sensitive (untreated) cells μ_s, we measure the fraction $\hat{P}$ of wells that

developed resistance before treatment initiation (i.e., during the expansion phase in absence of drug). Since the latters grow unperturbed when treatment is applied, the early-emerging resistant clones, arising within the first 3-4 weeks of drug exposure in our experimental setting represent the cells that developed resistance before treatment (see Extended data fig. 7) The first part of the MC-LD model uses an estimator for the mutation rate of sensitive (untreated) cells from the number of pre-existing mutants that were generated before the exposure to the drug. Following the approximations found in Eq (11) , for this case we have

$$\widehat{\mu_S} = -\frac{\log\left(1-\widehat{P}\right)}{\frac{1}{d}X(0)e^{(b-d)T_{treat}}\log\left(\frac{e^{-(b-d)T_{treat}}-b/d}{1-b/d}\right)}, \quad (12)$$

where $X(0)$ is the number of sensitive cells present in the population at the beginning of the fluctuation test. As described in the main text, during our fluctuation test sensitive cells were allowed to expand for a time T_{treat} before starting the treatment. The standard deviation of the estimated value of $\widehat{\mu_S}$ was obtained by error propagation of the standard deviation of the fraction of observed wells harboring resistant clones $\widehat{P}$, and is defined by the following expression [21]

$$\sigma_{\widehat{P}} = \sqrt{\frac{\widehat{P}\left(1-\widehat{P}\right)}{\#\ \text{total wells}}}. \quad (13)$$

The mutation rate Eq. (12) is a *chronological* rate, i.e. it quantifies the number of resistant cells emerging in the population per unit of time and per individual. As in this case new sensitive cells are generated by cell division, this rate can be converted into units of generation $\widehat{\mu_S} \rightarrow \widehat{\mu_S} t_{gen}$, where $t_{gen} = \frac{1}{b}$ is the duration of one generation. The values of the observed number of wells with pre-existing colonies of resistant cells are show in Fig. 3e, while the values of the inferred mutation rate are reported in Supplementary table 5.

Estimator of mutation rate for persister cells. To estimate the mutation rate of persister cells $\mu_p$in the second part of the MC-LD assay, we extended the experimental procedure described for sensitive cells, prolonging the treatment of persisters-containing wells and therefore performing a fluctuation assay which evaluates the fraction of the remaining wells developing resistant clones. As described above (Eq. 10), the expected fraction of resistant cells derived from persister cells and evaluation at time T is given by

$$P(T) = 1 - e^{-\mathcal{M}_p(T)}\,, \tag{14}$$

where

$$\begin{aligned} \mathcal{M}_p(T) &= \mu_p \int_0^T Z(t)\,\psi(T-t)dt \\ &= \mu_p\, X(0)\mathrm{e}^{(\mathrm{b}-\mathrm{d})\mathrm{T_{treat}}}\left(1-\frac{\mathrm{d}}{\mathrm{b}}\right)\left((1-f_0)\,\frac{\lambda([M])e^{S_0t_0}}{S([M])+Dp}\,\left(\frac{e^{S([M])(T-t_0)}-1}{S([M])}+\frac{e^{-D_p}-1}{D_p}\right)-\frac{f_0e^{S_0t_0}}{D_p}\left(e^{-D_p(T-t_0)}-1\right)\right). \end{aligned} \tag{15}$$

where $Z(t) = X(0)e^{(b-d)T_{treat}}z(t)$, is the number of persister cells expected at time t (product of the expected fraction of persister cells $z(t)$ from Eq.(5) and the total number of cells present in the wells at the beginning of treatment $X(0)e^{(b-d)T_{treat}}$), and we have used the approximation $\psi(\Delta t) \simeq \left(1-\frac{\mathrm{d}}{\mathrm{b}}\right)$. The estimator for the mutation rate of persister cells can be obtained by matching the expected probability (Eq. 14) to the observed fraction of wells with growing colonies observed $\hat{P}(T)$:

$$\widehat{\mu_p} = -\frac{\log\left(1-\hat{\mathrm{P}}(T)\right)}{X(0)\mathrm{e}^{(\mathrm{b}-\mathrm{d})\mathrm{T_{treat}}}\left(1-\frac{\mathrm{d}}{\mathrm{b}}\right)\left((1-f_0)\,\frac{\lambda([M])e^{S_0t_0}}{S([M])+Dp}\,\left(\frac{e^{S([M])(T-t_0)}-1}{S([M])}+\frac{e^{-D_p}-1}{D_p}\right)-\frac{f_0e^{S_0t_0}}{D_p}\left(e^{-D_p(T-t_0)}-1\right)\right)}, \tag{16}$$

Our analytcal derivation (Eq.s 14, 15 and 16) was validated with simulations of the MC-LD model, by testing the estimator (Eq. 16) with synthetic data (see Fig. 4c).

Bayesian inference of the mutation rate of persister cells. The mutation rate of persister cells was inferred with a Bayesian framework, in order to account for the uncertainty of the value of the initial fraction of persister cells, f_0. The bayesian inference of μ_p was obtained by fitting the MC-LD model expectation (Eq.s 14 and 15) to the observed fraction of wells with late-emerging resistant clones (evaluated at T=11 weeks for WiDr and at T=15 weeks for DiFi). The likelihood function was set to a standard Gaussian likelihood with mean equal to $\hat{P}(T)$ and standard devation $\sqrt{\frac{\hat{\mathrm{P}}(1-\hat{\mathrm{P}})}{\mathrm{W}}}$, where W is the number of wells that did not harbour early emerging resistant clones. The model expectation was computed using the model parameters inferred for the TP model (Supplementary table 4) . For the inference of the mutation rate of DiFi cl. B6, we used a value $t_0 = 0$, consistently with the data-set of Supplementary figure 4. We inferred two parameters: (i) the initial fraction of persister cells f_0 and (ii) the fold increase of the mutation rate of peristers cells vs sensitive cells $r = \frac{\mu_p}{\mu_s}$, i.e., the mutation rate was inferred a the product of this parameter and the inferred value of the mutation rate of sensitive cells, μ_s (see above). For the first parameter, f_0, we used an exponential prior distribution with the same mean as the one reported in Supplementary table 4, i.e., we imposed a prior on this parameter which is equal to the posterior distribution obtained when fitting the TP model to the growth curve data (Fig. 2d, Extended data fig. 8 and 9). For the second parameter, $r = \frac{\mu_p}{\mu_s}$ we used a flat distribution with minimum value 1 and maximum value 500. The posterior distributions for f_0 and for the fold increase of the mutation rate (shown in Fig. 4d , Extended data fig. 8 and 9) were sampled using a Hamiltonian Monte Carlo (HMC) algorithm (*Python 3*, package *pymc3*, NUTS sampler)[17] . Values of the estimated mutation rate and its standard deviation, reported in Supplementary table 5, were computed as $\mu_p = <r> \mu_s$, and $\sigma_{\mu_p} = \sigma_r \, \mu_s$, where $<r>$ and σ_r are the mean and the standard deviation of the posterior distribution of the parameter r.

Statistics & Reproducibility

No statistical method was used to predetermine sample size in the inference of the TP model parameters. The number of wells, the initial population size in each well and the time of cell replication in the absence of drug treatment were set by theoretical considerations using the MC-LD model, incorporating the population dynamics parameters that we previously measured (Supplementary tables 1 and 4). More in detail, assuming a mutation rate of about 10^{-9} mutations per generations/per individuals, we set these parameters by requiring that few (10-50) wells would generate resistant clones. In the following experiments, plates were checked to exclude from the analysis wells containing resistant clones, with the aim of characterizing cancer persister cells: *Single-dose* growth curve assay (Fig. 1d and Extended data fig. 1c); characterization of distribution of persister cells (Extended data fig. 6); staining with Carboxy fluorescein succinimidyl ester (CFSE) (Extended data fig. 3); EdU staining (Extended data fig. 3).

Supplementary Figures and Legends

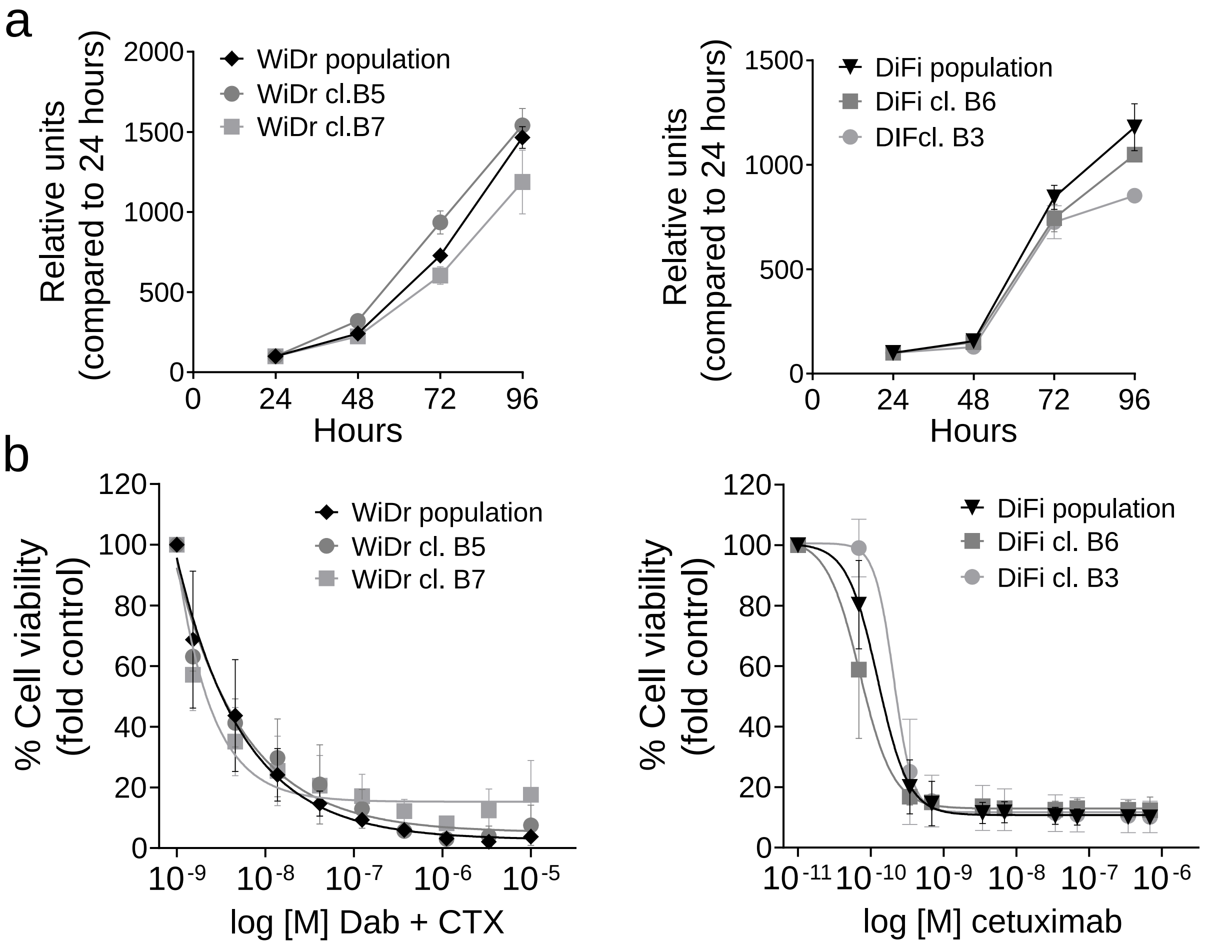

Supplementary figure 1. Growth kinetics of CRC cell populations and individual clones. a, The growth kinetics of WiDr and DiFi clones were compared with that of parental population at the indicated timepoints. Cell viability was measured by the ATP assay. **b**, The indicated cells were treated with increasing concentrations of dabrafenib (Dab) + 50µg/mL cetuximab (CTX) (WiDr) and increasing concentrations of cetuximab (DiFi). Cell viability was measured with the ATP assay after 5 (WiDr) or 6 days (DiFi). Results in both panels represent the average ± SD (n=3 biologically independent experiments).

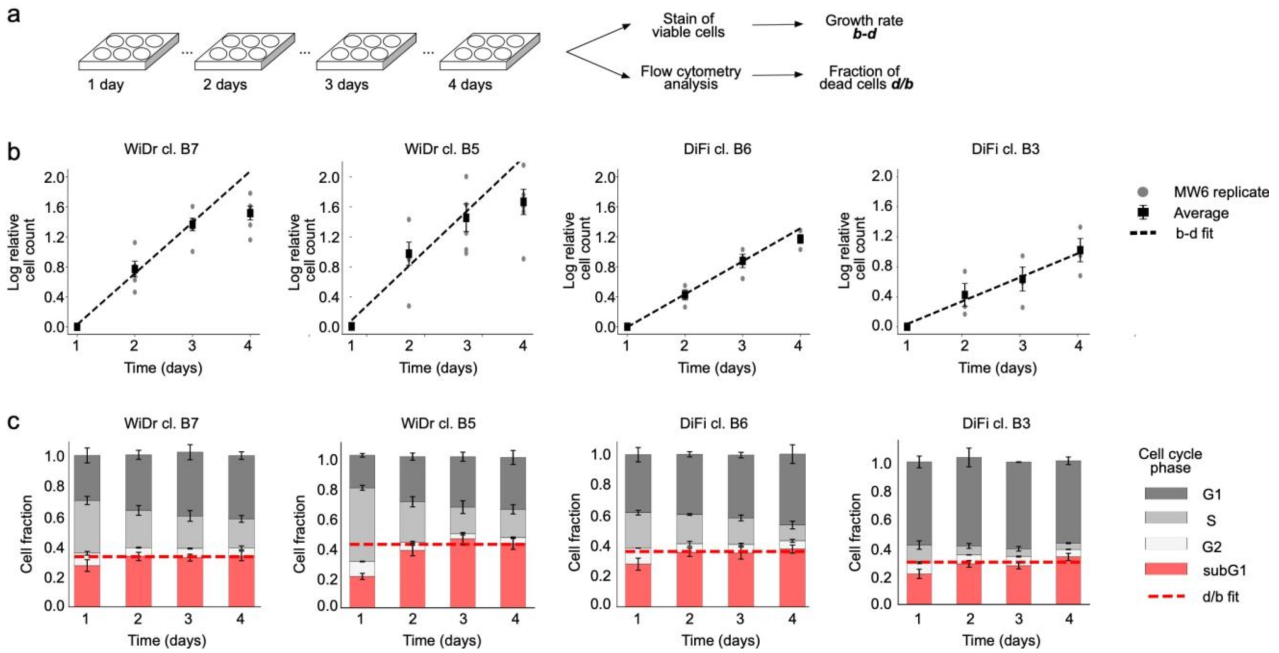

Supplementary figure 2. Birth and death rates of CRC cell clones. a, Schematic representation of the experimental setting used to evaluate birth and death rates of CRC cells. **b**, To establish the growth rate (*b*-*d*), the indicated cell models were seeded in multiple 6-multiwell plates (MW6) at 3.5-4x10^5 cells/well, and the number of viable cells was measured by manual count using trypan blue staining at the indicated time points. Grey dots represent individual biological replicates, each reported as mean of two technical replicates. Black squares represent average of biological replicates reported as mean ± SD (n=5 biologically independent experiments for WiDr cl. B7 and B5; n=3 biologically independent experiments for DiFi cl. B6 and B3). The black dashed line shows the best exponential fit (here represented as a linear fit on the log number of relative cell count). **c**, Cell cycle distribution of CRC clones measured by propidium iodide staining and flow cytometry analysis at the indicated time points. The fraction of cells in sub-G1 phase was used to estimate the death rate (*d/b*). Bars represent mean ± SD (n=3 biologically independent experiments). Red dashed line shows the best fit for the value *d/b*, which is the expected asymptotic value of the fraction of dead cells δ.

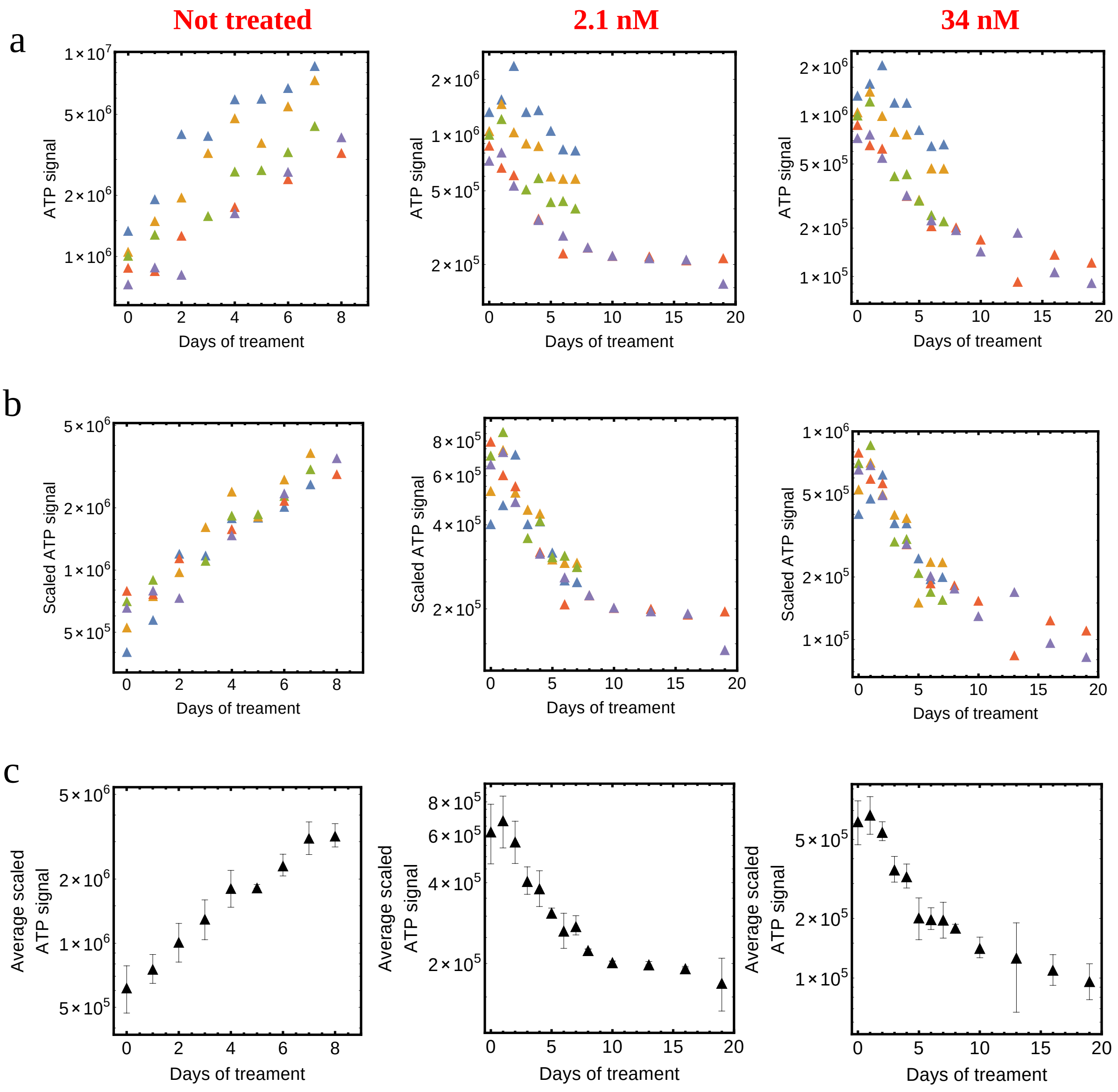

Supplementary figure 3. Illustration of the normalization process of the doses-response assay data used to obtain growth curves. a, Originally measured values of the cell viability, quantified by the ATP signal for DiFi cl. B6 cells. The plots report ATP signal vs days of treatment of 5 biological independent replicates (marked with different colors) and for the indicated drug concentrations. **b,** Values of the ATP signal shown in **a** are scaled by a constant value which is replicate-specific, i.e., it is the same for each data point collected in the same replicate (Supplementary methods section 'Calculation of growth curves from drug response assays'). **c** Values of the scaled ATP signal averaged across the 5 replicates and presented as mean ± standard deviation.

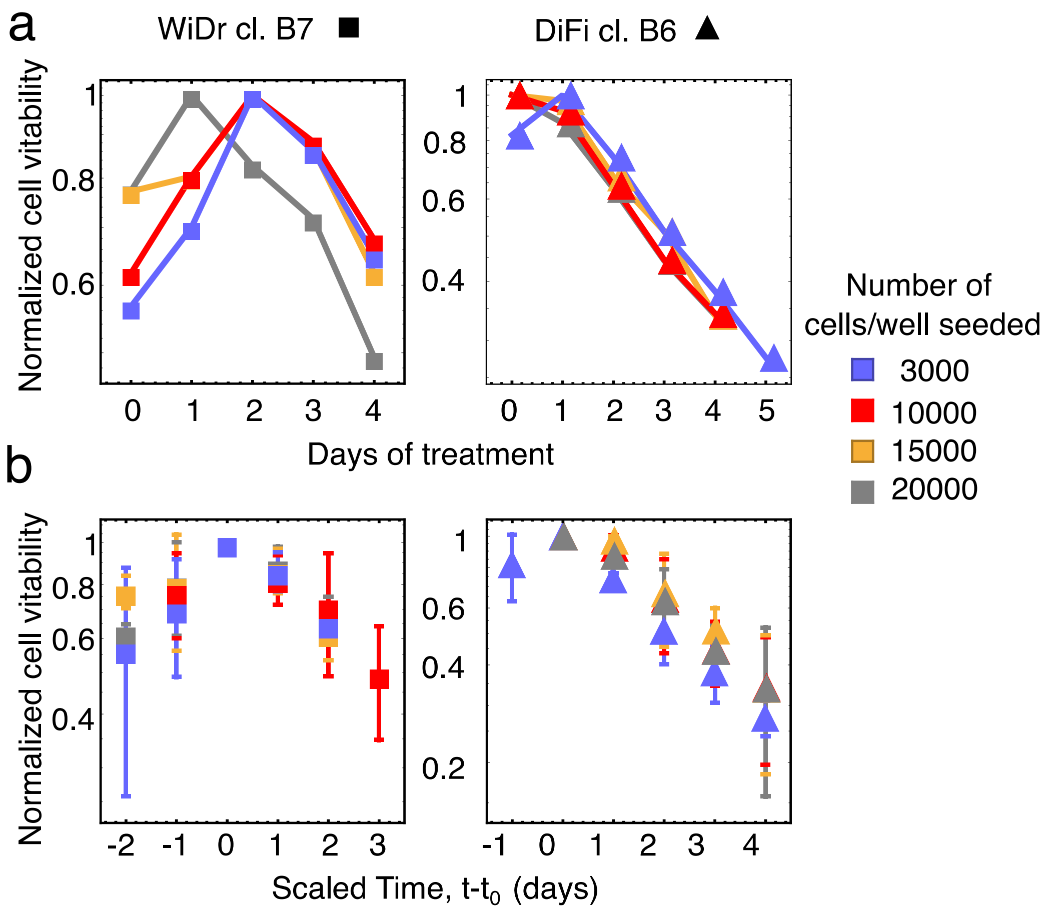

Supplementary figure 4. Impact of seeding density on cell growth dynamics. a, Cells seeded at indicated densities, were treated with 1µM dabrafenib + 50µg/ml cetuximab (WiDr) or 100 µg/ml cetuximab (DiFi). Cell viability was measured with the ATP assay at indicated time points of n=3 biologically independent experiments for both WiDr and DiFi. **b**, Cell growth assays performed with different initial number of cells display the same dynamics after an initial delayed effect of the treatment. All the values were normalized to the maximum value of cell viability measured. Time values from (**a**) were scaled to t_0 (time of delayed drug effect) indicating the time when the maximum cell viability was reached (n=3 biologically independent experiments for both WiDr and DiFi). Data are presented as mean values ± standard deviation.

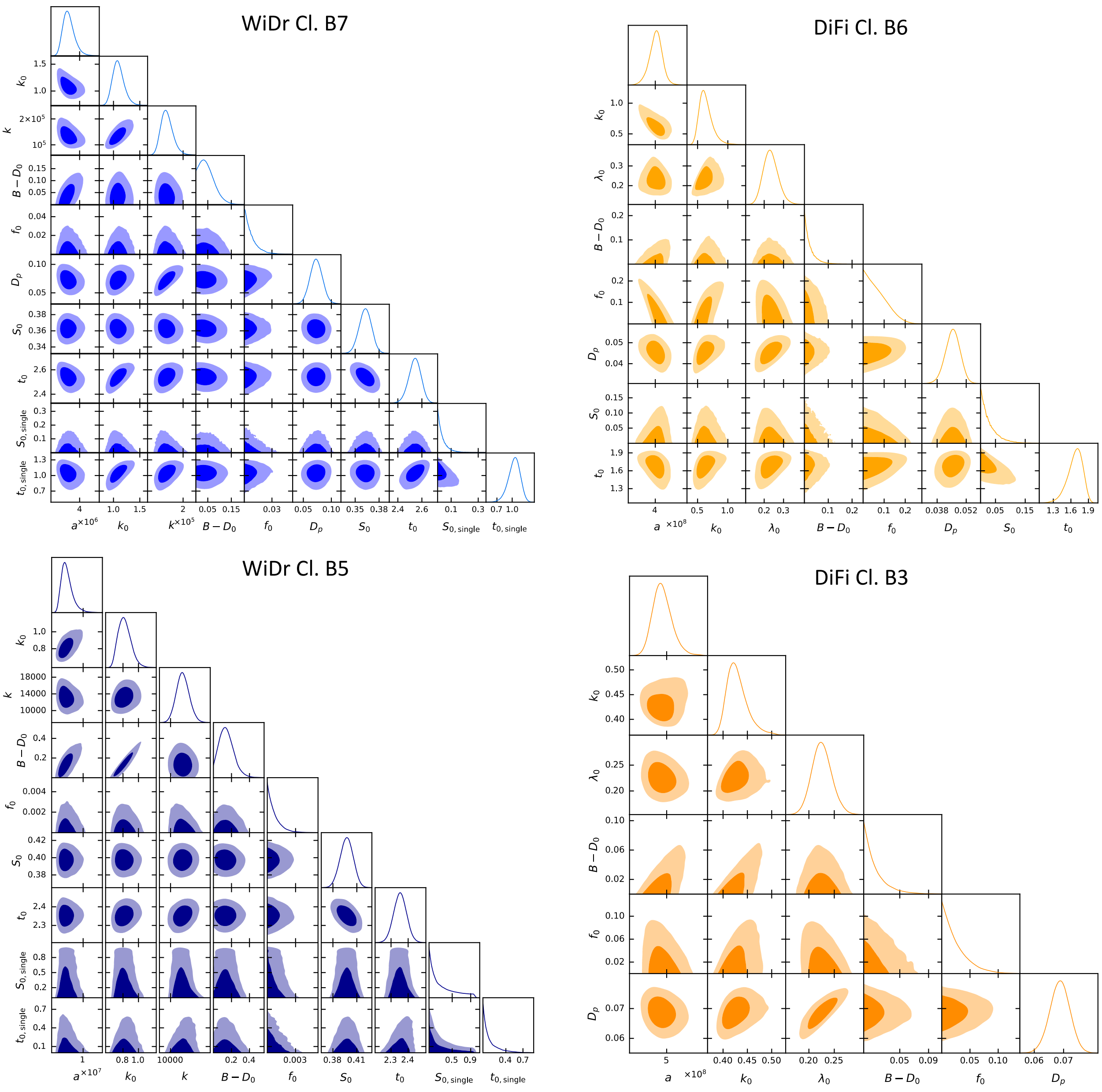

Supplementary figure 5. Posterior distributions of the inferred parameters for the transition to persister (TP) model. For each model we show posterior distributions of the inferred parameters. In the diagonal we show distributions marginalized to one parameter, off-diagonal plots show contour plots at one and two sigmas (dark and light orange, respectively) of distributions marginalized to two parameters. See Supplementary methods for description of Bayesian inference. The model parameters are the following: ***a:*** inverse of the characteristic drug concentration. For a drug concentration $[M] \ll a^{-1}$ cells die with their unperturbed death rate D_0, while for concentrations

$[M]>>a^{-1}$ the death rate κ reaches the maximal value ($D_0 + k_0$). $\boldsymbol{k_0}$**:** maximum death rate due to the drug. ***k*** coefficient of proportionality between drug concentration and rate of transition of sensitive cells to persister cells ($\lambda=k_0[M]$). $\boldsymbol{t_0}$***:*** time of the delay of the drug effect after administration. $\boldsymbol{B-D_0}$***:*** growth rate in absence of drug (birthrate *(B*) minus death rate D_0). $\boldsymbol{S_0}$: growth rate observed in the temporal window between drug administration and drug effect. $\boldsymbol{\lambda_0}$: rate of transition of sensitive cells to persister state. ***Dp***: death rate of persister cells. $\boldsymbol{t_{0\ (single)}}$***:*** delay in time of drug effect after treatment administration in the *single-dose assay*. $\boldsymbol{S_{0(single)}}$: observed growth rate in the time window between treatment administration and drug effect ($t<$ $\boldsymbol{t_{0(single)}}$) in the *single-dose assay*.

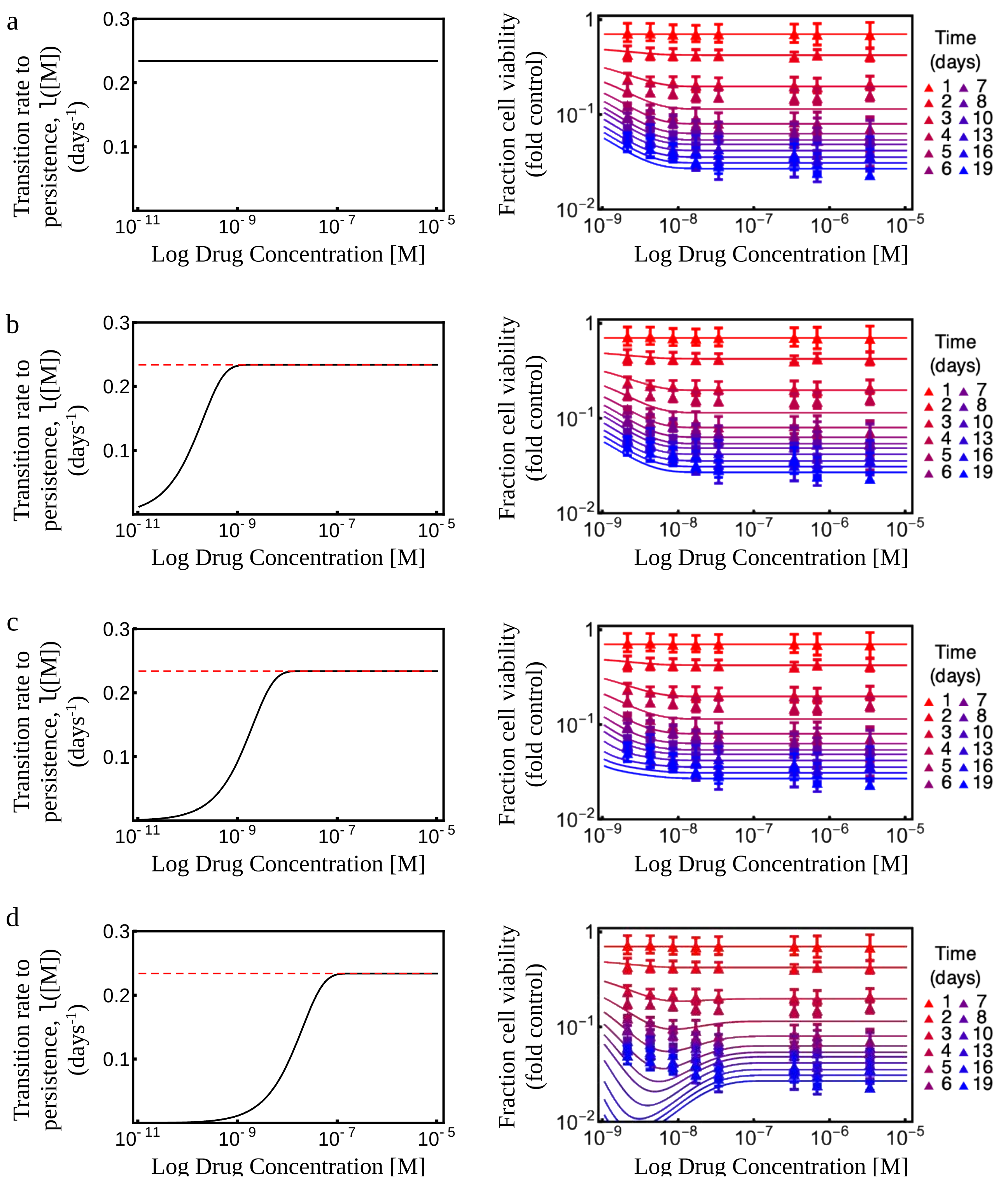

Supplementary figure 6. DiFi cl. B6 single-dose data are compatible with a TP model with drug-dependent transition rate. The figure shows the TP model fit (plots on the right-hand side) associated to four different scenarios (panels **a**, **b**, **c** and **d**) where the dependence on the drug concentration (black continuous lines in the left-hand side plots) differs. The first scenario (**a**) shows

the preferred TP model variant, a constant transition rate. In the other three scenarios (**b-d**), the functional dependence of the transition rate on the drug concentration was assumed to be $\lambda([M]) = \lambda_0 \, (1 - e^{-\beta[M]})$, which is such that the transition rate λ reaches a plateau for drug concentration $M \gg \beta^{-1}$. Second Column: best model fit associated to the specified drug dependence on the drug concentration (lines) shown together with the single dose data. Values of the parameters: **a,** λ_0=0.234 days^{-1}, **b,** λ_0 =0.234 days^{-1}, β=5x10^9 Mol^{-1}. **c,** λ_0 =0.234 days^{-1}, β =5x10^8 Mol^{-1}. **d,** λ_0 =0.234 days^{-1}, β =5x10^7 Mol^{-1}. The fits for the scenarios illustrated in panels **b** and **c** display a good agreement with the data, similarly to **a**, suggesting that DiFi cl. B6 can also be described by a drug-dependent transition rate. In all the panels we show data of n=5 biologically independent experiments and data are presented as mean values ± standard deviation.

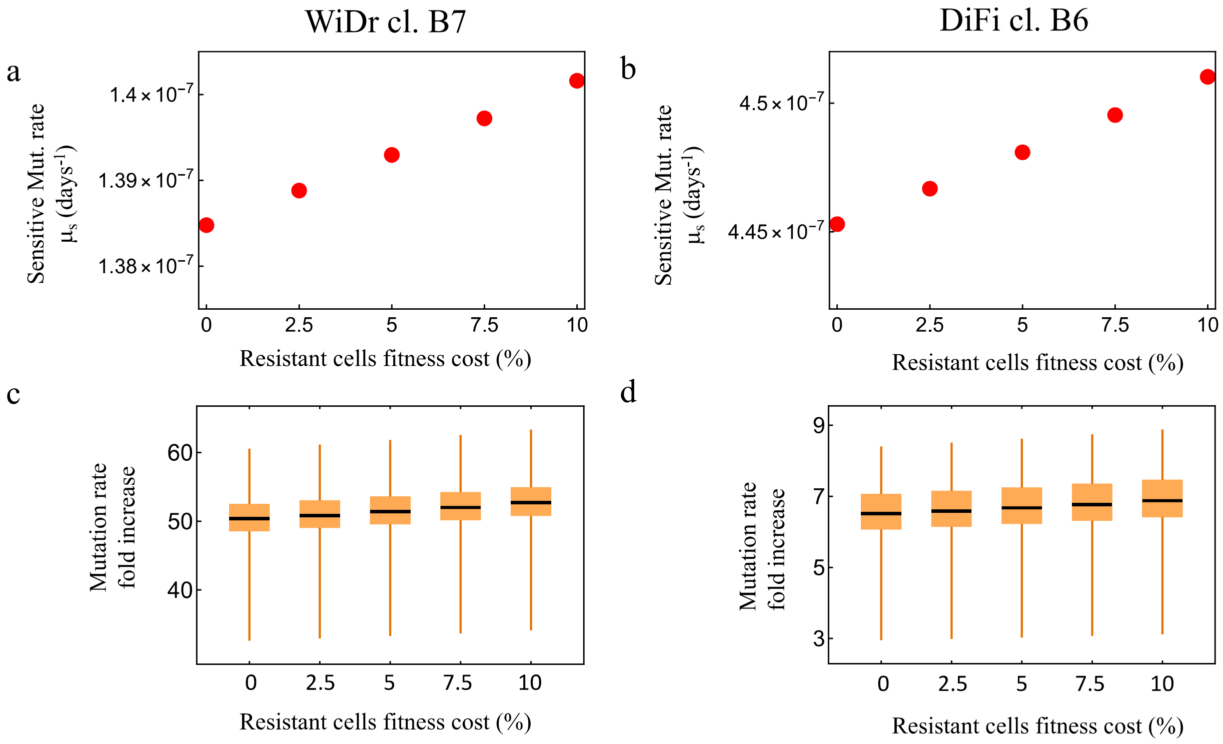

Supplementary figure 7. The inferred values of the mutation rate are stable against the variation of the value of the fitness cost of resistant cells. a-b, Inferred values of the mutation rate of sensitive cells as a function of the fitness cost of resistant cells for WiDr cl. B7 (**a**) and DiFi cl. B6 (**b**). The variation of the estimated mutation rates is very small. **c-d,** Inferred values of the fold increase of the mutation rate of persister cells with respect to sensitive cells as a function of the fitness cost of resistant cells for WiDr cl. B7 (**c**) DiFi cl. B6 (**d**). Shown here are results inferred form n=2 biologically independent experiments for both DiFi and WiDr. In the boxes shown in (c-d) the black line marks the mean value, boxes are for 25% and 75% percentiles, while fences show maximum and minimum value.

547

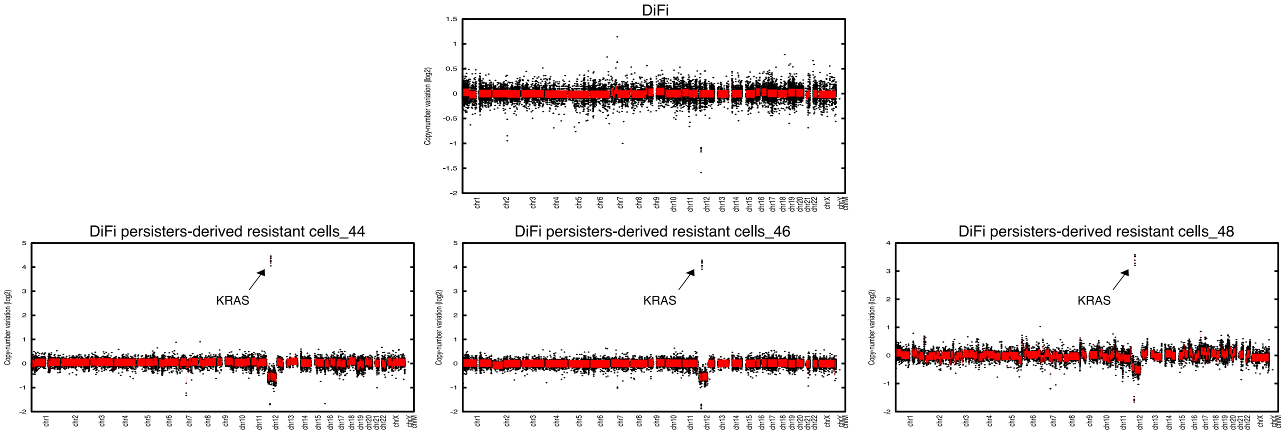

Supplementary figure 8. Gene copy number analysis of DiFi persisters-derived resistant cells. Copy number variation (CNV) analysis of DiFi resistant cells derived from persisters within the MC-LD experimental setting. Upper panel shows the CNV differential analysis of two different parental (sensitive untreated) clones. CNVs of three independent persister-derived resistant clones (namely clones 44, 46, 48) vs the parental counterpart are shown in the lower panels. Arrows indicate the KRAS amplification acquired at resistance to targeted therapy in all the 3 clones analyzed.

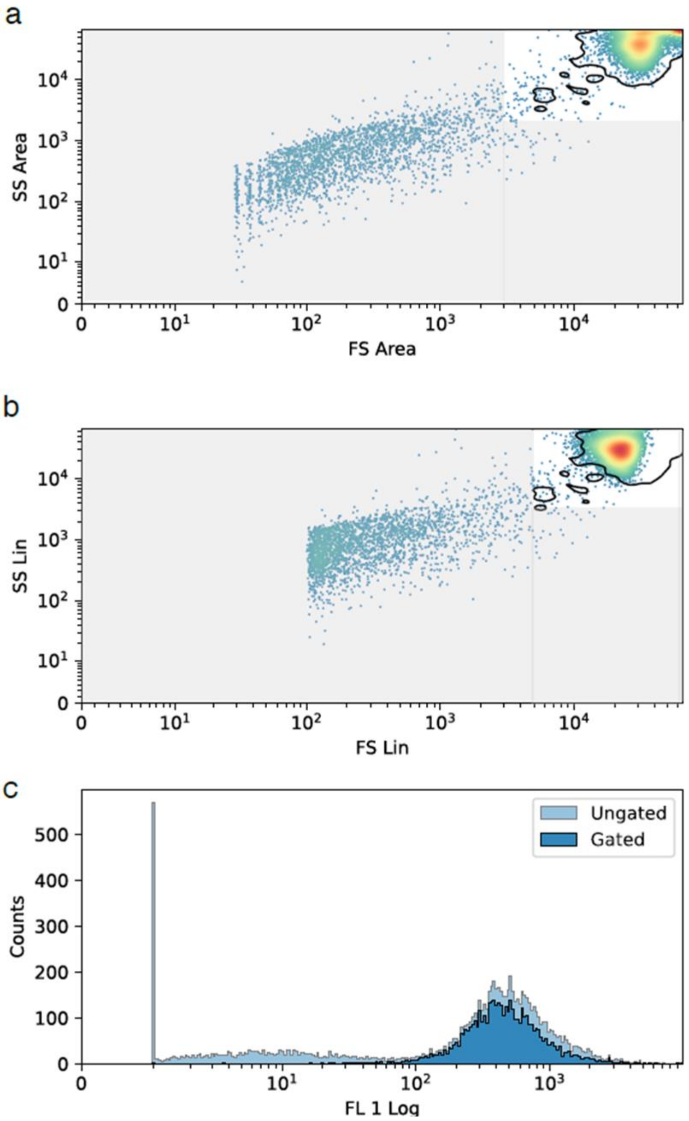

Supplementary figure 9. Example of the gating strategy used for CFSE data. Scatter plot of a typical CFSE dataset showing values of FSLin vs SSLin (**a**) and FSArea vs SSArea (**b**). The gating strategy consisted in applying the following cutoffs (marked in shadowed grey area in the scatter plots): (i) FS Lin: lower 5000 and upper 60000; (ii) SS Lin: lower 3000 and upper 63000; (iii) FS Area: lower 3000 and upper 60000; SS Area: lower 2000 and upper 63000. We then evaluated the bi-dimensional distribution of the remaining data points in the space of the coordinates FS Area and SS Area, and retained all the data-points that were included in the 99nth percentile of the distribution (area within the back line). **c,** Comparison of the distribution of Fitc signal for ungated *vs* gated cells.

Supplementary references

1. Russo, M. *et al.* Adaptive mutability of colorectal cancers in response to targeted therapies. *Science* **366**, 1473-1480 (2019).
2. Misale, S. *et al.* Emergence of KRAS mutations and acquired resistance to anti-EGFR therapy in colorectal cancer. *Nature* **486**, 532-6 (2012).
3. Prahallad, A. *et al.* Unresponsiveness of colon cancer to BRAF(V600E) inhibition through feedback activation of EGFR. *Nature* **483**, 100-3 (2012).
4. Kopetz, S. *et al.* Encorafenib, Binimetinib, and Cetuximab in *BRAF* V600E–Mutated Colorectal Cancer. *N Engl J Med* (2019).
5. Medico, E. *et al.* The molecular landscape of colorectal cancer cell lines unveils clinically actionable kinase targets. *Nature Communications* **6**(2015).
6. Brauner, A., Fridman, O., Gefen, O. & Balaban, N.Q. Distinguishing between resistance, tolerance and persistence to antibiotic treatment. *Nat Rev Microbiol* **14**, 320-30 (2016).
7. Vallette, F.M. *et al.* Dormant, quiescent, tolerant and persister cells: Four synonyms for the same target in cancer. *Biochem Pharmacol* **162**, 169-176 (2019).
8. Cipponi, A. *et al.* MTOR signaling orchestrates stress-induced mutagenesis, facilitating adaptive evolution in cancer. *Science* **368**, 1127-1131 (2020).
9. Di Nicolantonio, F. *et al.* Replacement of normal with mutant alleles in the genome of normal human cells unveils mutation-specific drug responses. *Proc Natl Acad Sci U S A* **105**, 20864-9 (2008).
10. Oren, Y. *et al.* Cycling cancer persister cells arise from lineages with distinct programs. *Nature* **596**, 576-582 (2021).
11. Sommer, C., Straehle, C., Köthe, U. & Hamprecht, F.A. Ilastik: Interactive learning and segmentation toolkit. in *2011 IEEE International Symposium on Biomedical Imaging: From Nano to Macro* 230-233 (2011).
12. Berg, S. *et al.* ilastik: interactive machine learning for (bio)image analysis. *Nat Methods* **16**, 1226-1232 (2019).
13. Crisafulli, G. *et al.* Whole exome sequencing analysis of urine trans-renal tumour DNA in metastatic colorectal cancer patients. *ESMO Open* **4**(2019).
14. Russo, M. *et al.* Reliance upon ancestral mutations is maintained in colorectal cancers that heterogeneously evolve during targeted therapies. *Nat Commun* **9**, 2287 (2018).
15. Gardner, S.N. A mechanistic, predictive model of dose-response curves for cell cycle phase-specific and -nonspecific drugs. *Cancer Res* **60**, 1417-25 (2000).
16. Gillespie, D.T. Stochastic simulation of chemical kinetics. *Annu Rev Phys Chem* **58**, 35-55 (2007).
17. Hoffman, M.D. & Gelman, A. The No-U-Turn Sampler: Adaptively Setting Path Lengths in Hamiltonian Monte Carlo. *Journal of Machine Learning Research* **15**, 1593-1623 (2014).
18. Lewis, A. GetDist: a Python package for analysing Monte Carlo samples. *arXiv astro-ph.IM*, 1910.13970 (2019).
19. Bailey, N.T.J. *The Elements of Stochastic Processes with Applications to the Natural Sciences*, (Wiley, 1990).
20. Van Kampen, N.G. *Stochastic Processes in Physics and Chemistry*, 480 (Elsevier Science, 1992).
21. Ycart, B. & Veziris, N. Unbiased estimation of mutation rates under fluctuating final counts. *PLoS One* **9**, e101434 (2014).
