# Supplementary material for: A modified fluctuation-test framework characterizes the population dynamics and mutation rate of colorectal cancer persister cells
Source: Nat Genet. 2022 Jul 11;54(7):976–84. doi: 10.1038/s41588-022-01105-z (PMC9279152; doi:10.1038/s41588-022-01105-z)
Supplement: Supplementary file 2 — Reporting summary [file 41588_2022_1105_MOESM2_ESM.pdf]

nature portfolio

Corresponding author(s): Prof. Alberto Bardelli and Prof. Marco Cosentino Lagomarsino.

Last updated by author(s): Apr 13, 2022

Reporting Summary

Nature Portfolio wishes to improve the reproducibility of the work that we publish. This form provides structure for consistency and transparency in reporting. For further information on Nature Portfolio policies, see our Editorial Policies and the Editorial Policy Checklist.

Statistics

For all statistical analyses, confirm that the following items are present in the figure legend, table legend, main text, or Methods section.

n/a	Confirmed	
☐	☒	The exact sample size (*n*) for each experimental group/condition, given as a discrete number and unit of measurement
☐	☒	A statement on whether measurements were taken from distinct samples or whether the same sample was measured repeatedly
☒	☐	The statistical test(s) used AND whether they are one- or two-sided<br>*Only common tests should be described solely by name; describe more complex techniques in the Methods section.*
☒	☐	A description of all covariates tested
☒	☐	A description of any assumptions or corrections, such as tests of normality and adjustment for multiple comparisons
☐	☒	A full description of the statistical parameters including central tendency (e.g. means) or other basic estimates (e.g. regression coefficient) AND variation (e.g. standard deviation) or associated estimates of uncertainty (e.g. confidence intervals)
☒	☐	For null hypothesis testing, the test statistic (e.g. *F*, *t*, *r*) with confidence intervals, effect sizes, degrees of freedom and *P* value noted<br>*Give P values as exact values whenever suitable.*
☐	☒	For Bayesian analysis, information on the choice of priors and Markov chain Monte Carlo settings
☒	☐	For hierarchical and complex designs, identification of the appropriate level for tests and full reporting of outcomes
☒	☐	Estimates of effect sizes (e.g. Cohen's *d*, Pearson's *r*), indicating how they were calculated

Our web collection on statistics for biologists contains articles on many of the points above.

Software and code

Policy information about availability of computer code

Data collection	Tecan SparkControl Magellan (v. 2.2); Summit 4.3.; Leica Application Suite Advanced Fluorescence (v. 2.6.3.8173); Nis-Element AR (v. 5.21.03 64 bit); QuantaSoft (v. 1.7.4.0917).
Data analysis	GraphPad prism (v.8); FlowJo (v.7.6); ImageJ (v. 1.53a); Adobe Photoshop CS5; Ilastik (v. 1.3.3 opensource); Matlab R2121a (The mathworks); Fiji 1.53 opensource; QuantaSoft (v. 1.7.4.0917); Microsoft Excel 2010; Python 3.9.7; Mathematica 10; C++14; g++ 10.3.0. Bioinformatic code for sequencing data are available at https://bitbucket.org/irccit/idea/src/master/; R (v. 3.4.4). Custom code used for the analysis are available as a repository on Mendeley Data (doi:10.17632/mvfm7hs9kw.1)

For manuscripts utilizing custom algorithms or software that are central to the research but not yet described in published literature, software must be made available to editors and reviewers. We strongly encourage code deposition in a community repository (e.g. GitHub). See the Nature Portfolio guidelines for submitting code & software for further information.

Data

Policy information about availability of data

All manuscripts must include a data availability statement. This statement should provide the following information, where applicable:

- Accession codes, unique identifiers, or web links for publicly available datasets
- A description of any restrictions on data availability
- For clinical datasets or third party data, please ensure that the statement adheres to our policy

Data used for the analysis, source data images of Edu staining and live microscopy assay are available as a repository on Mendeley Data (doi:10.17632/mvfm7hs9kw.1). Sequencing data are available at PRJEB49483 (ENA; https://www.ebi.ac.uk/ena/browser/home). The CRC cell clones generated in this study are available through Alberto Bardelli (Department of Oncology, University of Torino) under a Material Transfer Agreement.

Field-specific reporting

Please select the one below that is the best fit for your research. If you are not sure, read the appropriate sections before making your selection.

☒ Life sciences ☐ Behavioural & social sciences ☐ Ecological, evolutionary & environmental sciences

For a reference copy of the document with all sections, see nature.com/documents/nr-reporting-summary-flat.pdf

Life sciences study design

All studies must disclose on these points even when the disclosure is negative.

Sample size	We have used a model-guided approach combined to Bayesian statistics. Within this framework, we did not perform frequentist statistical tests; hence, no considerations on minimal sample size to ensure statistical significance were needed.<br>Nonetheless, the MC-LD model was used to estimate the sample size to be used during the fluctuation test assay, in order to assure that growing colonies of pre-existing resistant clones were likely to be found in the wells after four weeks. This prior knowledge was necessary to measure mutation rates of sensitive cells.
Data exclusions	In the following experiments:<br>- Single-dose growth curve assay (Fig. 1d)<br>- Characterization of distribution of persister cells (Extended data fig. 6)<br>- Staining with Carboxy fluorescein succinimidyl ester (CFSE) (Extended data fig. 3)<br>- EdU staining (Extended data fig. 3)<br>plates were checked to exclude from the analysis wells containing resistant clones, with the aim to characterize cancer persister cells.
Replication	The number of times each experiment has been repeated with similar results is stated in each figure legend or in the methods section.
Randomization	No randomization, e.g. into positive vs control samples, is applicable to our experimental designs, which are based on a model-guided approach and Bayesian statistics.
Blinding	Manual count of viable cells was assessed by two operators in blinded fashion.

Reporting for specific materials, systems and methods

We require information from authors about some types of materials, experimental systems and methods used in many studies. Here, indicate whether each material, system or method listed is relevant to your study. If you are not sure if a list item applies to your research, read the appropriate section before selecting a response.

Materials & experimental systems

n/a	Involved in the study	
☒	☐	Antibodies
☐	☒	Eukaryotic cell lines
☒	☐	Palaeontology and archaeology
☒	☐	Animals and other organisms
☒	☐	Human research participants
☒	☐	Clinical data
☒	☐	Dual use research of concern

Methods

n/a	Involved in the study	
☒	☐	ChIP-seq
☐	☒	Flow cytometry
☒	☐	MRI-based neuroimaging

Eukaryotic cell lines

Policy information about cell lines

Cell line source(s)	WiDr and DiFi CRC cell populations were obtained by Prof. Bernards and Prof. Baselga, respectively, as previously reported in our work "Medico et al., Nature Communications 2015". JVE207 CRC cells were obtained by Dr. Wezel, Department of Pathology, Leiden, University Medical Center.
Authentication	The identity of each cell line was checked no more than three months before performing the experiments using the PowerPlex® 16 HS System (Promega), through Short Tandem Repeats (STR) tests at 16 different loci (D5S818, D13S317, D7S820, D16S539, D21S11, vWA, TH01, TPOX, CSF1PO, D18S51, D3S1358, D8S1179, FGA, Penta D, Penta E, and amelogenin). Amplicons from multiplex PCRs were separated by capillary electrophoresis (3730 DNA Analyzer, Applied Biosystems) and analyzed using GeneMapper v.3.7 software (Life Technologies). STR results for all the cell lines and corresponding clones matched the profiles.
Mycoplasma contamination	Cells were routinely screened for absence of Mycoplasma contamination using the Venor® GeM Classic kit (Minerva biolabs) and tested negative.

Commonly misidentified lines
(See ICLAC register)

No commonly misidentified lines were used in this study.

Flow Cytometry

Plots

Confirm that:

☒ The axis labels state the marker and fluorochrome used (e.g. CD4-FITC).

☒ The axis scales are clearly visible. Include numbers along axes only for bottom left plot of group (a 'group' is an analysis of identical markers).

☐ All plots are contour plots with outliers or pseudocolor plots.

☐ A numerical value for number of cells or percentage (with statistics) is provided.

Methodology

Sample preparation

- Growth rates: CRC cells were fixed in EtOH 70% and stained with Propidium Iodide following manifacturer's instructions.
- CFSE staining: CRC cells were stained with CellTrace™ CFSE Cell Proliferation Kit (Invitrogen™) according to manufacturer's instructions. At the indicated timepoints, cells were collected and resuspended in 1mL PBS with Zombie Violet™ 1000x (BioLegend®) to exclude dead cells.

Instrument

Beckman Coulter CyAnTM ADP

Software

- Growth rates: Data were collected using Summit (v. 4.3) and analyzed using FlowJo (v. 7.6)
- CFSE staining: Data were collected using Summit (v. 4.3) and analyzed with a Python script based on standard libraries (FlowCal, FlowKit).

Cell population abundance

Sorting experiments were not performed

Gating strategy

- Growth rates: First gate: FSLin vs SSLin; Second gate: Pulse Width vs FL3Area

- CFSE staining: cells were selected with a light scattering gate (FSLin vs SS), excluding cell doublets with a single cell gate (FSArea vs SSArea). The following cutoffs were used: (i) FS Lin: lower 5000 and upper 60000; (ii) SS Lin: lower 3000 and upper 63000; (iii) FS Area: lower 3000 and upper 60000; SS Area: lower 2000 and upper 63000. We then evaluated the bi-dimensional distribution of the remaining data points in the space of the coordinates FS Area and SS Area, and retained all the data-points that were included in the 99nth percentile of the distribution. Viable cells were selected by excluding Zombie VioletTM-positive cells and CFSE signal was detected by measuring Fitc signal.

☒ Tick this box to confirm that a figure exemplifying the gating strategy is provided in the Supplementary Information.
